# Editorial: The complex interplay between mental health and extreme forms of violence- a critical perspective

**DOI:** 10.3389/fpsyt.2026.1795302

**Published:** 2026-02-26

**Authors:** Clare Sarah Allely, Angelo Zappalà

**Affiliations:** 1 School of Health and Society, University of Salford, Salford, United Kingdom; 2 CrimeLab, Istituto Universitario Salesiano Torino Rebaudengo (IUSTO), Torino, Italy; 3 CBT.ACADEMY, Torino, Italy; 4 Abo Akademi, Turku, Finland

**Keywords:** affective violence, extreme overvalued beliefs, predatory violence, school shooters, social isolation, mass shooting, school shooting, mass shooters

The relationship between mental illness and mass shootings, as well as with other forms of extreme violence (targeted violence, lone actor attacks, domestic terrorism), must be understood within a multifactorial model that avoids both stigma (“the diagnosis causes the attack”) and its denial (“mental health is irrelevant”). Meta-analyses indicate that certain severe disorders may be associated with an increased risk of violence, especially when co-occurring with substance abuse, history of aggression, environmental stressors, and active symptoms, while the majority of people with psychiatric diagnoses do not commit violent acts (1). In light of this evidence, it is important to move beyond simplistic assumptions and consider how contextual and methodological factors may shape both the observed association between psychiatric conditions and violence and the ways this association is measured and interpreted in specific phenomena such as mass shootings. Lankford and Cowan (2020) emphasized that the role of mental health issues in mass shootings may be underestimated not only for public health reasons (to avoid stigma and generalizations), but also due to methodological issues. Furthermore, evidence suggests that nearly all public mass shooters may have mental health problems, but social stigmas, which reduce the likelihood that perpetrators will seek psychological treatment, may help explain why the general public tends to underestimate this connection (2). However, it was observed that mass shootings and related forms of extreme violence cannot be reduced to a single causal factor, whether neurobiological, psychological, or social. The reviewed literature makes clear that a diagnosis of mental illness, on its own, cannot fully account for the phenomenon of mass shootings or other severe gun violence, nor can it reliably explain, predict, or prevent these events (3). Rather, they emerge from the interplay of individual vulnerabilities with broader contextual factors such as cultural scripts, stigma-related barriers to treatment, and access to lethal means.

This Research Topic brings together four complementary perspectives that advance our understanding of extreme violence through evolutionary, psychological, conceptual, and methodological lenses. Each contribution illuminates a distinct facet of this complex phenomenon while collectively pointing toward an integrated framework for assessment and prevention.

Meloy revisits the bimodal model of violence, distinguishing affective violence (reactive, emotion-driven, with intense autonomic arousal) from predatory violence (planned, low-arousal, goal-variable). The author presents ten normative criteria to differentiate these two modes and argues that this distinction is foundational for both retrospective forensic evaluation and prospective threat assessment, as it guides data interpretation, diagnosis, prognosis, and risk management. These ten criteria include: Intense autonomic arousal vs. minimal autonomic arousal; subjective experience of emotion vs. no conscious emotion; reactive violence vs. planned, purposeful violence; perceived threat vs. no perceived threat; goal is threat reduction vs. variable goals; possible displacement vs. no displacement of the target; time limited vs. no time limitations; preceded by public posturing vs. private ritual; altered awareness vs focused awareness and emotional defense vs. cognitive attack.

Lankford and Silva investigated the impact of social isolation among public mass shooters. Specifically, they compared public mass shooters who were socially isolated (n = 56) and those public mass shooters who were not socially isolated (n = 67) in the United States from 2000 to 2024. Their findings suggest that socially isolated perpetrators tend to experience greater difficulties than other mass shooters in terms of mental health and life circumstances (e.g., be unemployed; have a history of suicidality which is not related to their planned attack; have a diagnosis of autism spectrum disorder and have mental health difficulties), are more likely to engage in certain unhealthy behaviors (e.g., substance abuse; demonstrate an interest in previous acts of mass violence), and are more prone to carry out more destructive attacks. As highlighted by the authors, these findings are associations only as causation cannot be tested. Lankford and Silva argue that the findings from this study indicate the presence of some important causal pathways and they propose a new model to explain social isolation’s effects on public mass shooters.

Kristinsdottir et al. address conceptual fragmentation through a bibliometric analysis of publications on extreme, overvalued, and delusion-like beliefs. Their review reveals significant terminological inconsistency: psychiatry emphasizes diagnostic categories (delusions, schizophrenia), while social sciences focus on terrorism and radicalization. The concept of extreme overvalued beliefs—shared beliefs that are absolute, emotionally charged, and resistant to contradicting evidence—emerges as particularly relevant for understanding fixated offenders who lack frank psychosis yet demonstrate pathological commitment to violence. Unlike delusions, extreme overvalued beliefs align with personality and are reinforced by online subcommunities. Distinguishing these cognitive-affective drivers of fixation is essential for forensic evaluation and treatment planning. The review by Kristinsdottir et al. indicated that the content of hateful or violent beliefs and ideologies that the extent to which the individual fixates on them is also important to consider in the monitoring of this behavior not just the content. Therefore, it is important to monitor and assess not just that the beliefs of the individual but how they believe them.

Minelli et al. provide a systematic review of sociodemographic and psychological characteristics of school shooters in the United States. Their most striking finding is the sheer extent of missing data: most characteristics they sought to analyze simply were not reported in the existing literature. Despite these gaps, some recurrent features emerged, including social isolation, bullying victimization, depression, and family dysfunction. The authors emphasize that school shooters are not a homogeneous group—demographic, experiential, and psychological differences suggest multiple underlying profiles rather than a single perpetrator typology. They call for standardized data collection protocols to overcome the fragmentation that currently impedes systematic understanding of the phenomenon.

Understanding the bimodal model of violence and exploring the impact of social isolation; extreme, overvalued, and delusion-like beliefs; sociodemographic and psychological characteristics in perpetrators of extreme acts of violence (e.g., mass shootings, targeted violence, lone actor attacks, domestic terrorism) are important for informing assessment, and intervention. Also, for informing violence prevention. For instance, the study by Lankford and Silva emphasizes the importance of individuals creating meaningful social connections in their lives. There is a role for government organizations, community-based organizations, media and entertainment corporations, technology companies, public health professionals, schools, workplaces, and families for increasing meaningful social connections.

Collectively, the contributions in this Research Topic converge on a central insight: extreme violence cannot be adequately understood, assessed, or prevented through any single disciplinary lens. Whether examining the neurobiological underpinnings of predatory versus affective aggression, the social dynamics of isolation and fixation, or the cognitive architecture of extreme overvalued beliefs, each article demonstrates that reductive explanations are insufficient and that meaningful progress requires integrating individual-level vulnerabilities with contextual, relational, and methodological considerations. At the same time, Minelli et al. documentation of pervasive data gaps serves as a sobering reminder that the field’s capacity for evidence-based prevention remains constrained by the fragmentation and inconsistency of its empirical foundations. Moving forward, advancing both research and practice will require not only multidisciplinary collaboration among clinicians, researchers, and policymakers, but also a commitment to standardized assessment frameworks and data collection protocols that can support cumulative knowledge-building across diverse forms of extreme violence.
